# Beyond type 1 vs. type 2 processing: the tri-dimensional way

**DOI:** 10.3389/fpsyg.2014.00993

**Published:** 2014-09-09

**Authors:** Alexandra L. Varga, Kai Hamburger

**Affiliations:** Experimental Psychology and Cognitive Science, Justus Liebig UniversityGiessen, Germany

**Keywords:** dual-process theories, goals, closed-world reasoning, cognitive economy, theoretical model

## Type 1 and type 2 processing—the dichotomy, the continuum, and a tentative third way

**Figure 1:**
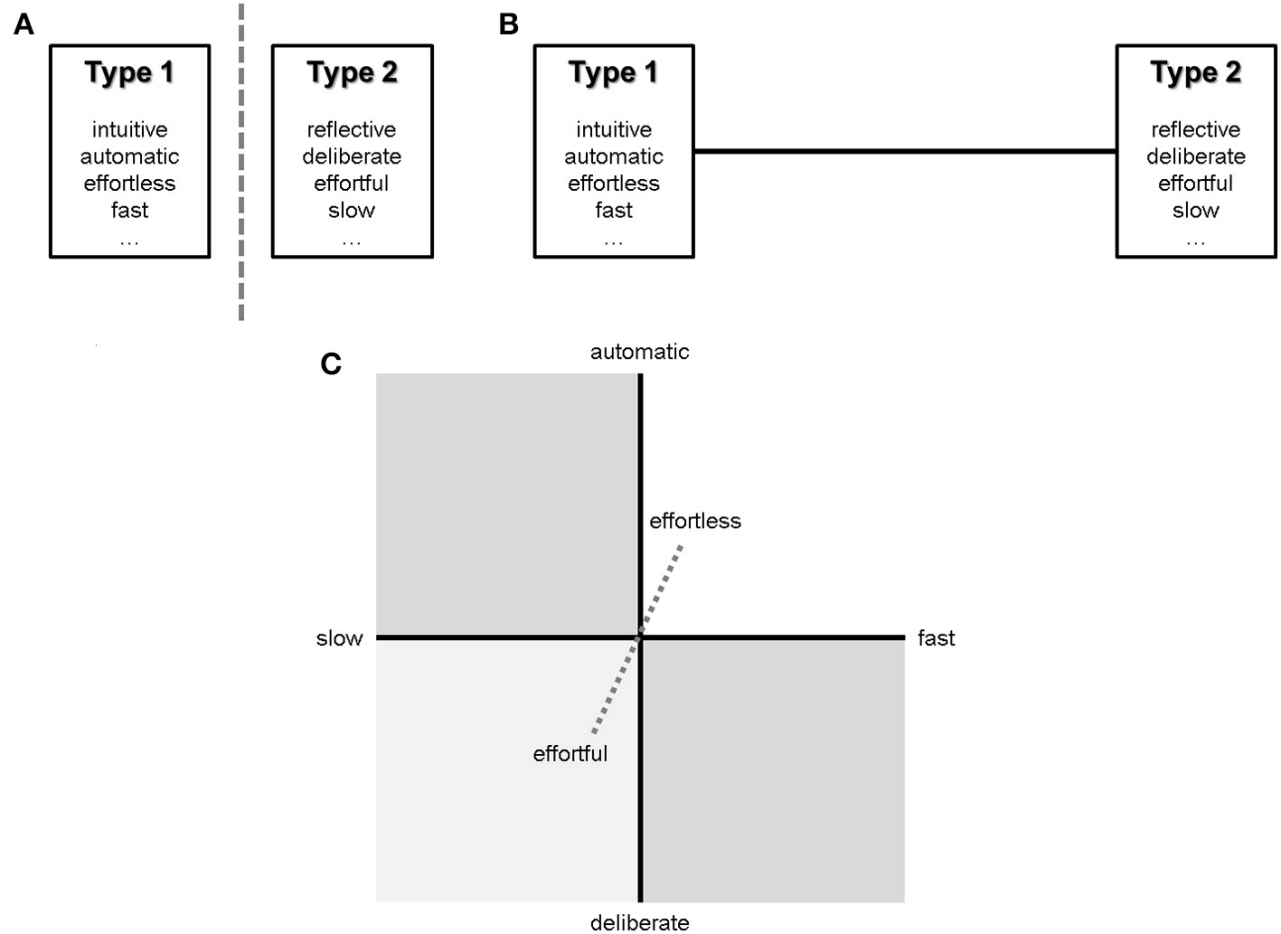
**(A)** Type 1 and type 2 processes - the dichotomy; **(B)** Type 1 and 2 processes - the continuum; **(C)** Tri-dimensional processing.

Dual-process theories have dominated social and cognitive psychology since the 1970's (Wason and Evans, 1975). The tenets have gone through many changes over time. This in mind, the theoretical core amounts to a dichotomous view of two types of processes (Figure 1A): type 1—intuitive, fast, automatic, nonconscious, effortless, contextualized, error-prone, and type 2—reflective, slow, deliberate, cogitative, effortful, decontextualized, normatively correct^1^. The received view (Evans, 2012) of dual-process theories is evaluative ^2^; Type 2 is better: it focuses on generalizable logical form, it is more productive, and closer to normative standards. However, Evans (2012) acknowledges that unambiguous categorization into either type is contentious. In the face of empirical and conceptual arguments that the emphasized distinctions are rather quantitative than qualitative (Osman, 2013), or that both modes of thought are underpinned by common mechanisms (Colder, 2011), the dichotomy has given way to a continuous view (Kruglanski and Gigerenzer, 2011; Kruglanski, 2013)—Figure 1B. One desirable effect of the continuum is an integrated view of cognition, which affords unified modeling of complex phenomena (argument from parsimony). Nonetheless, it too is problematic. First, it calls upon the need to fixate a turning point along the continuum. Second, manifold phenomena may be difficult to classify despite the linear relaxation of the dichotomy.

We tentatively propose a third way, whereby processing features are represented in a tri-dimensional space (Figure 1C). It uses three directly operationalizable, continuous dimensions from the received view of dual-processes theories: cognitive speed, effort, and control. “Cognitive effort” is the degree of utilization of mental resources, namely working memory, and computational capacity. “Control” is a dimension ranging from automatic to deliberate processes (Shiffrin and Schneider, 1977). We conjecture that the hierarchy of processing goals situates cognitive phenomena in the tri-dimensional model; processing features are goal-relative.

Because it represents processes as combinations of features, our proposal accommodates a broader range of phenomena without the Procustian pitfall of fitting them in a dichotomous pattern. Acknowledging the possibility of complex, effortful though automatic processes, for instance, the proposal is more attuned to recent findings that high-level cognition is not necessarily deliberate (e.g., Day and Gentner, 2007). Upon formal implementation, it affords integrated modeling of complex phenomena, thus safeguarding the argument from parsimony. Moreover, it downplays the tendency for fallacious (Evans, 2012) evaluative categorization of types of information processing.

## Illustrating tri-dimensional processing

Two main factors modulate processing features: practice, and goals.

Computer scientist Raymond Kurzweil states that “Predicting the future is actually the primary reason that we have a brain.” (Kurzweil, 2013; p. 31). We would add the reason to change the future according to our desires. The crucial reason for information processing is the ability to interact with the surrounding and to change it according to our purposes. We have the poly-faceted brains that we do for contextually optimal pursuit of the goals that call for our actions.

It is widely accepted that practice leads to better results, faster, with less effort (Shiffrin and Schneider, 1977). Practice is just the tip of the iceberg, since “a person's repertoire of strategies may depend upon many factors, such as cognitive development, experience, or formal education” (Payne et al., 1993; p. 33). Nonetheless, people typically engage in automatic, effortless, fast processing on familiar tasks, while relying on effortful and slow deliberation for novel ones (Monsell, 2003). The axes in Figure 1C are traversed through practice left-to-right and bottom-up. Because practice strengthens goals—sub-goals (means) associations (Rosenbloom and Newell, 1986; Duncan, 1990), we propose to capture its effects on processing in terms of goals. Well-practiced actions become defaults for achieving their goals. Novelty is thus a contextual characteristic that activates different goals or means.

Consider the cognitive processes that underpin driving, a complex behavior pursuing several sub-goals toward the overarching goal of reaching the destination. Let us examine how goals modulate processing features. Novices must control every step, e.g., attend to the gear lever while switching gears instead of watching the street. Each sub-goal is a task on its own. Overall, information processing is slow, effortful, and deliberate. With practice the sub-goals are integrated automatically and with less effort. Expert drivers *normally* process and perform faster the required actions.

Consider the case of a Frenchman driving in London with the overarching goal to reach TATE Modern. Although integration of the usual sub-goals, e.g., switch gears without attending the lever, is fast, the novel sub-goals, e.g., look right first, call for effortful deliberate processing. Now consider a driving instructor, i.e., a driver pursuing the additional main goal to teach. She must verbalize her steps such that her pupil understands and executes correctly. The pedagogical goal requires controlled deliberation. Nonetheless procedural prior knowledge of driving allows fast, effortless processing. Such examples are difficult to categorize as either type 1 or type 2 processing, while their tri-dimensional position is rather straightforward.

Generally speaking, when action consistency must be preserved across a different range of goals compared with default contexts, information processing is more effortful, slow and deliberate—the price of flexible thought. However, the underlying well-practiced processes remain automatic. We propose that deliberate control of automatized behaviors in novel situations is top-down, from goal setting to motor commands. The goal-centered analysis affords fine-grained categorization of complex processes, and thereby overcomes the “turning point” issue of continuous dimensions.

## Toward a cognitive model

The tri-dimensional view is amenable to a realistic process model. We follow Kruglanski and Gigerenzer's (2011) proposal that information processing is generally underpinned by rules. Additionally, we propose that rules, including those that ground automatic processes, can be represented logically.

“The individual's representation will generally be selective and will not include all features […]” (Payne et al., 1993; p. 21). Consider a rule prescribing ignorance of potential exceptions until explicit evidence is encountered: if there is no positive information about a given event, assume it does not occur. Those “given events” are abnormalities with respect to a habitual process, e.g., both additional goals from Section Illustrating Tri-Dimensional Processing define abnormalities relative to default driving contexts. Abnormalities can be represented as activations of different goals in non-default circumstances. Novel tasks are paradigmatically abnormal relative to familiar ones.

This rule is the closed-world assumption for reasoning about abnormalities (CWAab); it has been shown to be involved in various cognitive phenomena, e.g., the suppression-task (Stenning and van Lambalgen, 2008). CWAab promotes minimal cognitive effort, so it appropriately represents automatic processing. However, explicit evidence for abnormalities, e.g., everybody driving on the right, allows contextually overriding the assumption and hence flexible processing.

Closed-world reasoning is embedded in Logic Programming, a cognitively relevant formalism for backwards reasoning from goals to actions (Stenning and van Lambalgen, 2008). Information processing with CWAab is implementable in a Logic Programming cognitive model.

## The müller-lyer illusion—two-sided error source

Daniel Kahneman (2011) exemplifies the evaluative contrast of perceptual and cognitive phenomena. The Müller-Lyer illusion instantiates the correct type 2 vs. the illusory type 1 processing^3^.

“Now that you have measured the lines, you – your System 2, the conscious being you call “I”– have a new belief: you *know* that the lines are equal […] But you still *see* the bottom line as longer. You have chosen to believe the measurement, but you cannot prevent System 1 from doing its thing; you cannot decide to see the lines as equal, although you know they are. To resist the illusion […] you must learn to mistrust your impressions of the length of lines when fins are attached to them. To implement that rule, you must be able to recognize the illusory pattern and recall what you know about it. If you can do this, you will never again be fooled by the Müller-Lyer illusion. But you will still see one line as being longer than the other.” (Kahneman, 2011; p. 27)

In contrast, we argue that the Müller-Lyer figure can illustrate a perceptual (type 1) but also a cognitive illusion (type 2). When familiar with the illusion, I *know* that the lines' relative lengths are not as I *perceive* them. However, a substantial number of undetectable variations in length are possible. For instance, if the line perceived as longer in the original figure is elongated by 5% (below threshold, cf. Weber's law), physics and perception are congruent. In this case type 1 processes would provide the correct view, while type 2 would erroneously doubt this judgment because it “knows that it knows better.” Thus, systematic mistrust of type 1 processing is inappropriate.

Cognitive economy considerations raise further concerns. Kahneman has been one of the first to acknowledge such considerations while, however, attaching negative connotations to our profound “laziness” (Kahneman, 2011). We propose that always doubting our initial percept produces a costs—benefits imbalance, due to cognitive overload, and “paralysis in reasoning” or the inability to act. Under real time constraints it is often more costly to “check back” through deliberate reflection. *Normally* the automatic decisions and default actions are appropriate, i.e., they serve their purpose most efficiently^4^. A similar argument against the “systematic mistrust” of fast automatic processes has been endorsed by the ABC group. The mistrust is based on a “more is better” ideology, which “ignores the ecological rationality of cognitive strategies” (Gigerenzer et al., 1999; p. 20).

Consequently we view the Müller-Lyer figure as evidence that both type 1 and type 2 processes are error-prone. Therefore error-proneness is not a suitable criterion to categorize information processing. A model based on operationalizable processing features promises a more constructive approach, through predictions with respect to the kinds and amounts of errors, be those perceptual illusions or cognitive biases. The good and the bad are to be found a posteriori, through empirical work.

## Wrapping-up and further-on

Our proposal provides a more fine-grained analysis than the dichotomous dual-process theories: even complex processing tokens can be categorized along three continuous dimensions via teleological assessment. Processing types may become available a posteriori, if clusters arise in the tri-dimensional space. The goal-centered analysis helps to overcome the turning point issue of continuous dimensions. Regarding evaluative claims, we conclude that good reasoning is the balance point between efficient and flexible processing in a tri-dimensional space. “Goodness” is relative to currently activated goals, not an intrinsic feature of processing types.

We aim to corroborate the conjectures set forth in Sections Illustrating Tri-Dimensional Processing and Toward A Cognitive Model by constructing a tri-dimensional model of closed-world processing (e.g., in Logic Programming), testing predictions derived from it, and consequentially refining the proposed conceptualization.

### Conflict of interest statement

The authors declare that the research was conducted in the absence of any commercial or financial relationships that could be construed as a potential conflict of interest.

## Footnotes

^1^The relation between perceptual and affective, on the one hand, and cognitive processes, on the other hand, is, mutatis mutandis, analogous with that between type 1 and type 2 reasoning.

^2^Sloman (1996) is a notable exception.

^3^Due to space constraints, throughout this section we use type 1/2 as umbrella-terms for the features individuated in our tri-dimensional proposal.

^4^The CWAab prescribes these behaviors.
